# Violacein induces p44/42 mitogen-activated protein kinase-mediated solid tumor cell death and inhibits tumor cell migration

**DOI:** 10.3892/mmr.2015.3525

**Published:** 2015-03-20

**Authors:** TORAL MEHTA, KOEN VERCRUYSSE, TERRANCE JOHNSON, ANTHONY OKECHUKWU EJIOFOR, ELBERT MYLES, QUINCY ANTOINE QUICK

**Affiliations:** 1 Department of Biological Sciences, Tennessee State University, Nashville, TN 37209, USA; 2 Department of Chemistry, Tennessee State University, Nashville, TN 37209, USA

**Keywords:** violacein, apoptosis, migration, poly(ADP ribose) polymerase, p44/42

## Abstract

Microbial secondary metabolites have emerged as alternative novel drugs for the treatment of human cancers. Violacein, a purple pigment produced by *Chromobacterium violaceum*, was investigated in the present study for its anti-tumor properties in tumor cell lines. Clinically applicable concentrations of violacein were demonstrated to inhibit the proliferative capacity of tumor cell lines according to a crystal violet proliferation assay. The underlying mechanism was the promotion of apoptotic cell death, as indicated by poly(ADP ribose) polymerase cleavage and p44/42 mitogen-activated protein kinase signaling determined by western blot analysis. Collectively, this provided mechanistic evidence that violacein elicits extracellular-signal regulated kinase-induced apoptosis via the intrinsic pathway. The anti-malignant properties of violacein in the present study were further demonstrated by its inhibitory effects on brain tumor cell migration, specifically glioblastomas, one of the most invasive and therapeutically resistant neoplasms in the clinic. Additionally, solid tumors examined in the present study displayed differential cellular responses and sensitivities to violacein as observed by morphologically induced cellular changes that contributed to its anti-migratory properties. In conclusion, violacein is a novel natural product with the potential to kill several types of human tumor cell lines, as well as prevent disease recurrence by antagonizing cellular processes that contribute to metastatic invasion.

## Introduction

The utility of bacteria as agents for the treatment of cancer was described over a century ago and continues to be investigated for their therapeutic value as delivery agents for anti-cancer drugs and vectors for gene therapy (1–3). Additionally, contemporary strategies have also studied the use of bacterial products, including proteins, enzymes, immunotoxins and secondary metabolites for their anti-tumor properties (1–3). An advantage of these alternatives is the lack of systemic infection associated with the use of live, attenuated and engineered bacterial strains for cancer therapy (1–3).

In the present study, the secondary metabolite violacein, a pigment produced by the bacteria *Chromobacterium violaceum*, was investigated as an anti-tumor agent, which has been shown to have medicinal applications as an antibiotic and anti-trypanosoma agent (4,5). The use of bacterial metabolites such as violacein as anti-cancer agents is supported by pre-clinical studies with other microbial products, including farnesyltransferase inhibitors (6–14), prodigines (15–17) and epothilones (18–22), which have displayed positive therapeutic efficacy in several types of cancer and were subsequently examined in phase I and II clinical trials. More specifically, violacein has been shown to have anti-cancer properties in leukemia (23,24) and colon cancer cells (25–27), as well as in Ehrlich ascites tumors by Bromberg *et al* (28) using an *in vivo* mouse model. In contrast to these studies, the present study used violacein extracted from a *Chromobacterium violaceum* strain native to a copper basin in Tennessee, which was demonstrated here to have anti-proliferative effects on cell lines derived from solid tumors, as well as displayed an inhibitory effect on cancer cell migration, extending the anti-tumorigenic properties of this bacterial-produced metabolite.

## Materials and methods

### Cell conditions and reagents

U87 (glioblastoma), A549 (lung) and MCF7 (breast) cancer cells were purchased from the American Type Culture Collection (Manassas, VA, USA). All cell lines were maintained in Dulbecco’s Modified Eagle’s Medium (DMEM; Invitrogen Life Technologies, Carlsbad, CA, USA) containing 10% fetal bovine serum (Invitrogen Life Technologies), 2 mM L-glutamine (Invitrogen Life Technologies), 100 nM MEM non-essential amino acids (Invitrogen Life Technologies) and penicillin-streptomycin (Invitrogen Life Technologies) at 37°C and 5% CO_2_.

### Isolation and characterization of violacein

*Chromobacterium violaceum* was collected from environmental soil and water samples at the Tennessee Copper Mining Company site (Ducktown, TN, USA), also termed the Copper Basin. *Chromobacterium violaceum* were inoculated in Luria-Bertani broth (LB) media, streaked and grown on agar plates for 48 h at 30°C, until colonies formed. The colonies were subsequently selected and grown as cultures in LB broth media for 24–48 h at 30°C. The cultures were used to extract and purify violacein, as described previously (29). Cultures were centrifuged at 14,629 × g in 100% ethanol for 15 min at 4°C and the supernatant was collected, and violacein was separated and extracted using chloroform. The violacein was allowed to dry for 24 h and dissolved in 50% ethanol (Fisher Scientific, Fair Lawn, NJ, USA). Violacein was subsequently purified with reverse phase column chromatography and characterized by liquid chromatography-mass spectrometry and ultraviolet-visible spectroscopy.

### Crystal violet cell proliferation assay

Cells were plated in 24-well plates, treated with 250 nM, 500 nM and 1 *μ*M violacein and allowed to incubate for 48 h ([vehicle controls were treated with dimethyl sulfoxide (DMSO; Amresco, LLC, Solon, OH, USA)] for dose-response experiments. For time-course experiments, cells were treated with 1 *μ*M violacein and allowed to incubate for 1, 3 and 5 days. Subsequently, tissue culture medium was removed, the cell monolayer was fixed with 100% methanol for 5 min and stained with 0.5% crystal violet in 25% methanol for 10 min. Cells were then washed three times for 5 min each with distilled water to remove excess dye and allowed to dry overnight at room temperature. The incorporated dye was then solubilized in 0.1 M sodium citrate (Sigma-Aldrich; St. Louis, MO, USA) in 50% ethanol. Next, 100 *μ*l treated and control samples were transferred to 96-well plates and optical densities read at 540 nm using an X-mark microplate absorbance spectrophotometer (Bio-Rad, Hercules, CA, USA).

### Cell motility

Motility assays were conducted according to the manufacturer’s instructions (Cell Biolabs Inc., San Diego, CA, USA). A cell suspension containing 0.5–1.0×10^6^ cells/ml was prepared in serum-free media with vehicle (DMSO) or 1 *μ*M violacein, while 500 *μ*l of media containing 10% fetal bovine serum was added to the lower chamber of the migration plate. 300 *μ*l of the cell suspension containing vehicle or 1 *μ*M violacein was then added to the inside of each insert and allowed to incubate for 24 hours at 37°C and 5% CO_2_. Subsequently, non-migratory cells were removed from the plate inserts (per manufacturer’s instructions), migratory cells were counterstained, solubilized, and optical density densities read at 595 nm using a X-mark microplate absorbance spectrophotometer (Bio-Rad Laboratories).

### Western blot analysis

Cells were plated and treated with 1 *μ*M violacein for 24 h or vehicle (DMSO), rinsed with phosphate-buffered saline (Bio-Rad Laboratories, Hercules, CA, USA), and lysed with CelLytic M Cell lysis reagent (Sigma-Aldrich, St Louis, MO, USA). Protein concentrations were subsequently determined using the Bradford reagent (cat. no. B6916; Sigma-Aldrich). Proteins were separated by 8% SDS-PAGE (Bio-Rad Laboratories) and then transferred to nitrocellulose membranes. For immunoblotting, nitrocellulose membranes were incubated with phosphorylated (p) Akt (rabbit monoclonal; cat. no. 4606), cleaved poly(ADP ribose) polymerase (PARP; rabbit polyclonal; cat. no. 9544), p44/42 (rabbit monoclonal; cat. no. 4370), and pS6-ribosomal protein (rabbit monoclonal; cat. no. 4858) and β-actin (rabbit polyclonal; cat. no. 4967), purchased from Cell Signaling Technology (Danvers, MA, USA) and all diluted 1:25, recognizing target proteins overnight at 4°C. The membranes were then incubated with a horseradish peroxidase-conjugated goat anti-rabbit secondary antibody (1:100; cat. no. 7074; Cell Signaling Technology) for 3 h at room temperature, analyzed using an enhanced chemiluminescence detection system with SuperSignal West Pico Chemilluminescent Substrate (cat. no. 34080; Thermo Fisher Scientific, Waltham, MA, USA) and visualized by autoradiography using a BioSpectrum UVP Imaging System (BioSpectrum, Upland, CA, USA). Actin was used as loading control.

### Statistical analysis

Values are expressed as the mean ± standard error. Significance was determined using Student’s t-test. P<0.05 was considered to indicate a statistically significant difference between values.

## Results

### Violacein antagonizes brain, lung and breast cancer cell proliferation

**Figure 1:**
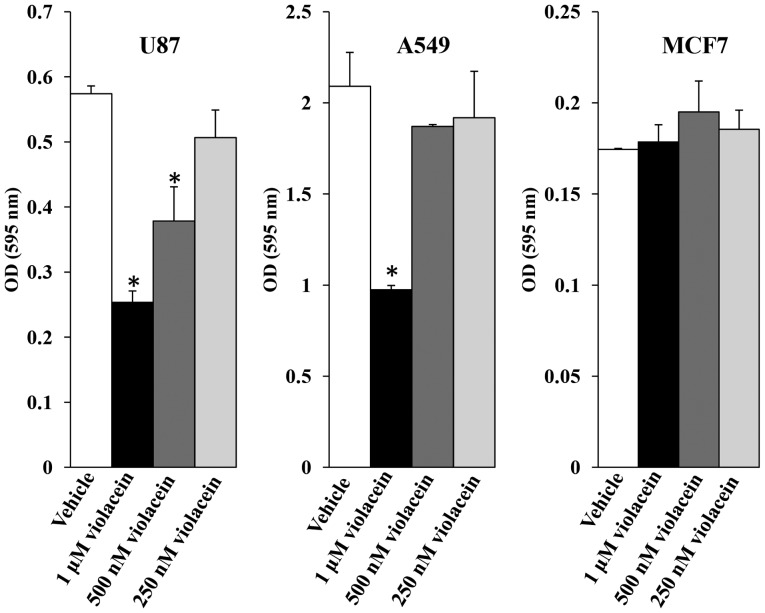
Dose response and sensitivity of solid tumor cell lines to violacein. Violacein caused a significant decrease in cell viability of U87 brain tumor cells and A549 lung cancer cells in contrast to MCF7 breast cancer cells. Shown is a dose-response experiment performed in duplicate representative of three independent experiments that showed similar results. White bars, vehicle dimethyl sulfoxide; black bars, 1 *μ*M violacein; dark grey bars, 500 nM violacein; light grey bars, 250 nM violacein. Error bars represent the standard error. ^*^P<0.05 compared to vehicle-treated control cells.

Lung and breast cancer are two of the most common types of cancer found in men and women, and have high incidence of metastasis to the brain (30). The present study therefore evaluated the effects of violacein on three solid tumor cell lines, U87 (brain), A549 (lung) and MCF7 (breast). Dose-response experiments were performed at the outset to assess the effects of several violacein concentrations (250 nM, 500 nM and 1 *μ*M) on cell viability. Cell viability dose-response assays revealed a 56-, 34- and 12%-decrease in U87 cells treated with 1 *μ*M, 500 nM and 250 nM violacein as compared to the viability of vehicle treated control cells, respectively, while A549 cells displayed a 54, 11 and 8%-decrease in viability when treated with identical concentrations compared to that of vehicle-treated control cells (Fig. 1). By contrast, MCF7 cell viability did not decrease in response to violacein exposure using this assay.

**Figure 2:**
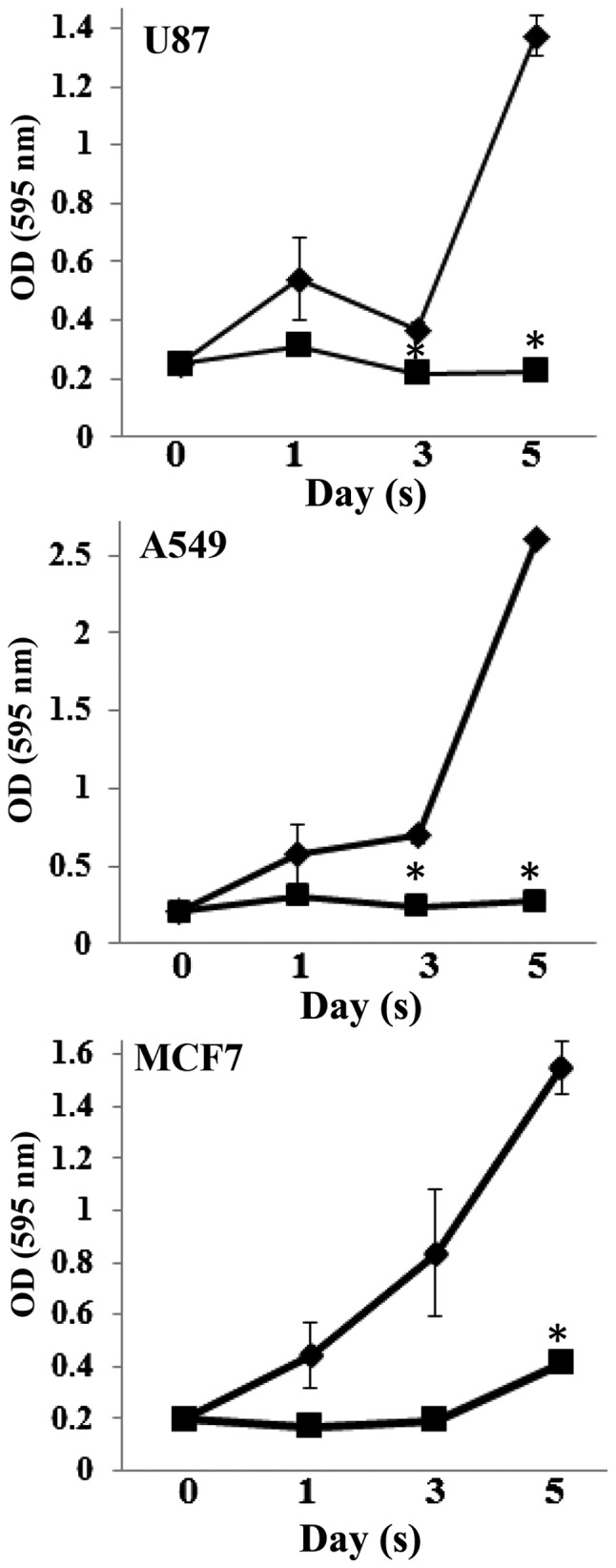
Anti-proliferative effects of violacein on cancer cells. The mitotic capacity of U87 brain tumor and A549 lung cancer cells was inhibited in response to treatment with 1 *μ*M violacein, while violacein decreased the proliferative rate of MCF7 breast cancer cells. Values are representative of three independent experiments performed in duplicate that showed similar results. Diamonds, vehicle dimethyl sulfoxide; squares, 1 *μ*M violacein. Error bars represent the mean±standard error. ^*^P<0.05 compared to vehicle-treated control cells at the corresponding time-point. OD, optical density.

The anti-proliferative effects of violacein have been attributed to its ability to elicit a cytotoxic response in several human cancers (23,27,31–33), as well as induce differentiation, a cytostatic response observed in leukemia cells by Melo *et al* (26). To gain better insight into the effect of violacein on cell proliferation in brain, lung and breast cancer cells, time course experiments were preformed in the present study. Examination of U87, A549 and MCF7 cells treated with 1 *μ*M violacein revealed a reduction in cell proliferation of all three cell lines over a five-day period, with a statistically significant difference (P<0.05) in cell viability observed on day five between vehicle-treated control cells and violacein-treated cells (Fig. 2). Time course analysis also revealed that U87 and A549 cells were more sensitive to violacein treatment as compared to MCF7 cells, which displayed a two-fold increase in cell viability five days post-violacein treatment as compared to cell viability on day 0 (Fig. 2). These data were consistent with dose response experiments that also showed that U87 and A549 cells were more sensitive to violacein exposure than MCF7 cells. The findings of the present study parallel a study by Menezes *et al* (34), which showed that crude extracts of violacein were differentially toxic to several human tumor cell lines, including the multi-drug-resistant ovarian tumor cell line NCI/ADR-RES.

### Violacein promotes PARP and p44/42 mitogen-activated protein kinase (MAPK) signaling

**Figure 3:**
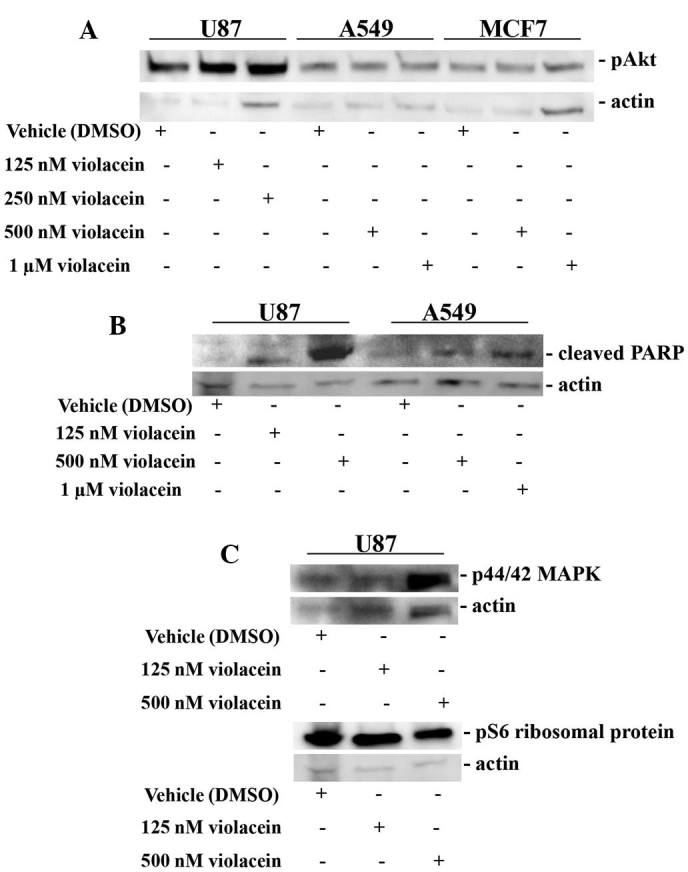
Regulation of survival and apoptotic signaling proteins by violacein. (A) Violacein had no effect on pAkt protein levels in solid tumor cell lines examined, (B) but substantially induced expression of the pro-apoptotic protein cleaved PARP in brain and lung cancer cells. (C) Additionally, violacein upregulated p44/42 MAPK protein levels in brain tumor cells. Immunoblots displayed are representative of at least three independent experiments that showed comparable results.

The mechanistic effects of violacein on cellular responses have proven to be varied and tumor type-specific. Of note, violacein has been shown to upregulate p53, p27 and p21, which are negative regulators of cell cycle progression (24), and to induce reactive oxygen species-mediated apoptotic cell death in colon cancer cells (23). Additionally, Ferreira *et al* (27) demonstrated that violacein promoted leukemia cell death via tumor necrosis factor signaling. To address the mechanisms of the anti-proliferative response of violacein on solid tumor cells described above, several intracellular signaling proteins were analyzed for changes in their expression when exposed to various concentrations of this agent. The survival and pro-apoptotic proteins, Akt and PARP, were examined in U87, A549 and MCF7 cells exposed to violacein. A substantial induction of cleaved PARP was observed in U87 brain tumor cells treated with 500 nM violacein, as well as in A549 lung cancer cells treated with 1 *μ*M violacein (Fig. 3). By contrast, no changes in the activity of Akt expression were observed in cells exposed to violacein (Fig. 3). Additional protein expression analysis revealed that 500 nM violacein upregulated p-44/42 MAPK in U87 cells. This result was similar to that observed in leukemia cells, which displayed increased p38 MAPK expression in response to violacein (27). Furthermore, violacein did not affect the expression levels of pS6 ribosomal protein (Fig. 3), providing additional evidence along with the lack of expressional changes of pAkt protein described above, that this secondary metabolite does not mechanistically influence the translational control signaling network.

### Impairment of tumor cell migration in response to violacein

**Figure 4:**
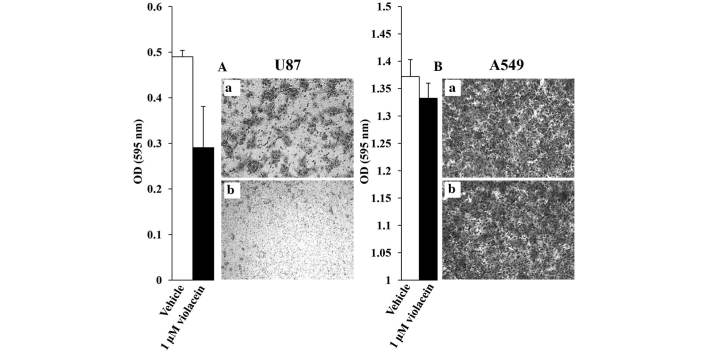
Effects of violacein on tumor cell migration. Violacein (A) considerably reduced brain tumor cell migration and (B) had a diminutive effect on lung cancer cell migration. Shown are boyden chamber assays performed in duplicate that are representative of three independent experiments that exhibited similar results (magnification, 40×). Aa and Ba, cells treated with vehicle dimethylsulfoxide; Ab and Bb, cells treated with 1 *μ*M violacein. OD, optical density.

**Figure 5:**
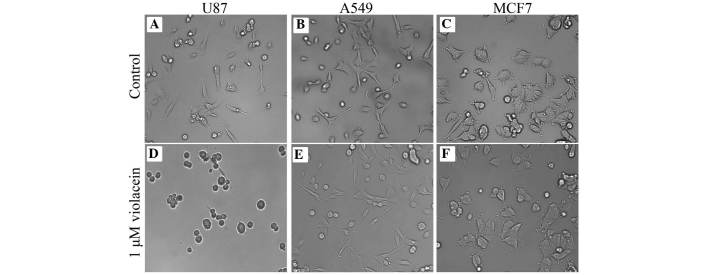
Violacein induces morphological changes that affect tumor cell motility. (A-C) Cells treated with vehicle dimethylsulfoxide. (D) U87 brain tumor cells displayed a transient round cellular phenotype in response to treatment with 1 *μ*M violacein, that was absent in (E) lung and (F) breast cancer cells. Micrographs shown are representative of five different experiments that showed similar results (magnification, 100×).

The effect of violacein on cellular biological processes that underlie metastatic invasion has not been sufficiently investigated to date, to the best of our knowledge. In the present study, the ability of violacein to inhibit cellular processes that contribute to the metastatic invasion of cancers, which leads to therapeutic resistance and recurrence of this disease, were therefore evaluated. Boyden chamber assays revealed that 1 *μ*M violacein decreased the migration of U87 cells by 40% as compared to that of vehicle-treated control cells, while a diminutive decrease was observed in A549 violacein-treated cells (Fig. 4). The anti-migratory response, particularly of U87 cells, may be attributed in part to violacein-induced morphological changes (Fig. 5). U87 cells exposed to 500 nM violacein appeared to detach from the cell substratum and exhibited a round cellular phenotype three hours post-exposure, which was not observed in A549 and MCF7 cells after treatment with violacein (Fig. 5). However, cell adhesion assays performed revealed that violacein did not affect the adhesive properties of U87 brain tumor cells (data not shown). It should also be mentioned that U87 cells re-attached and displayed normal cell morphology when examined 24 h after violacein exposure, indicating the violacein-induced morphological changes were not a consequence of cells undergoing cell death. In conclusion, these results suggested that violacein-induced morphological changes had an impact on the migratory ability of U87 brain tumor cells.

## Discussion

The efficacy of therapeutic agents used to treat human cancers is dependent upon their ability to antagonize molecular signaling events that contribute to the survival of tumor cells and induce regulatory mechanisms that promote their death. The present study investigated a novel secondary metabolite, violacein, produced by a chromobacterium, for its utility as an agent that can promote tumor cell death in solid tumor-derived cell lines. It was shown that violacein considerably reduced the proliferative capacity of lung and brain cancer cells, and to a much lesser extent that of breast cancer cells, providing an indication that cancers are differentially sensitive to this agent. This difference in sensitivity and responsiveness was also manifested mechanistically as demonstrated in the ability of violacein to promote apoptotic cell death by upregulating cleaved PARP, a downstream target of the effector pro-apoptotic molecule caspase 3, in brain and lung cancer cells. Additionally, p44/42, a known apoptosis-promoting regulator and caspase 3 activator, was increased in brain tumor cells treated with violacein. These results established that violacein-induced apoptosis observed in the present study likely occurs via the intrinsic pathway, particularly in brain tumor cells, which is consistent with studies on leukemia, fibrosarcoma and colon cancer cells that have demonstrated the apoptosis-promoting properties of violacein by intrinsic as well as extrinsic pathways (23,27,35). It should also be noted that violacein has been described to elicit other types of cell death, as shown in resistant leukemia cells that underwent cell death as a consequence of endoplasmic reticulum stress and breakdown of the golgi apparatus, underscoring the tumor cell killing capacity of violacein via different cell death mechanisms (25).

Although several studies, including the present study, have provided evidence of violacein’s ability to promote tumor cell death, its role as an agent that can prevent the metastatic invasion of cancer cells has received little attention. The present study showed that violacein inhibited brain tumor cell migration, likely as a consequence of disrupting sub-cellular domain structures of the actin filamentous network, including the lamellipodia and filopodia, that led to a round cellular phenotype that compromised the motility of these cells. To the best of our knowledge, the present study was the first to show that violacein inhibits cancer cell migration, extending the anti-malignant properties of this agent. Additionally, the anti-migratory effects of violacein on cancer cells demonstrated in the present study are further supported by a recent study by Platt *et al* (36), which showed that violacein inhibited the secretion of the pro-cell migratory inflammatory chemokine CXCL12 in breast cancer cells. These results suggested that violacein may have therapeutic applications that prevent brain tumor cells from invading normal brain tissue, as well as inhibit brain metastases from breast cancer, the most common source of metastatic brain tumors in women (30).

In conclusion, violacein has potential as a therapeutic agent to treat cancer due to its versatility to cause cell death in several types of cancer and prohibit metastatic invasion. The potential of violacein to be used as a cancer drug is further supported by the recent U.S. Federal Drug Administration’s approval of romidepsin, a histone deactylase inhibitor produced by *Chromobacterium violaceum*, for the treatment of T-cell lymphoma (37), as well as a renewed interest in cancer therapies utilizing bacteria and bacterial products (1–3) to selectively target cancer cells.
